# Adrenocortical Carcinoma (ACC) Cells Rewire Their Metabolism to Overcome Curcumin Antitumoral Effects Opening a Window of Opportunity to Improve Treatment

**DOI:** 10.3390/cancers15041050

**Published:** 2023-02-07

**Authors:** Marta Claudia Nocito, Paola Avena, Lucia Zavaglia, Arianna De Luca, Adele Chimento, Tarig Hamad, Davide La Padula, Davide Stancati, Constanze Hantel, Rosa Sirianni, Ivan Casaburi, Vincenzo Pezzi

**Affiliations:** 1 Department of Pharmacy and Health and Nutritional Sciences, University of Calabria, 87036 Arcavacata di Rende (CS), Italy; 2 Department of Endocrinology, Diabetology and Clinical Nutrition, University Hospital Zurich and University of Zurich, 8091 Zürich, Switzerland; 3 Medizinische Klinik und Poliklinik III, University Hospital Carl Gustav Carus Dresden, 01307 Dresden, Germany

**Keywords:** adrenocortical carcinoma, curcumin, ERRα, glutamine, metabolism

## Abstract

**Simple Summary:**

Curcumin is one of the primary ingredients in turmeric and curry powders. Therapeutic benefits of curcumin have been demonstrated in multiple diseases including cancer. In this study we show that curcumin reduces growth and migration of adrenocortical carcinoma (ACC), a very aggressive tumor. Additionally, curcumin changes ACC cell metabolism toward glycolysis and glutamine utilization. Our data point to curcumin combinational therapy with glutamine metabolism inhibitors as promising therapeutic intervention against ACC.

**Abstract:**

Extensive research suggests that curcumin interferes with multiple cell signaling pathways involved in cancer development and progression. This study aimed to evaluate curcumin effects on adrenocortical carcinoma (ACC), a rare but very aggressive tumor. Curcumin reduced growth, migration and activated apoptosis in three different ACC cell lines, H295R, SW13, MUC-1. This event was related to a decrease in estrogen-related receptor-α (ERRα) expression and cholesterol synthesis. More importantly, curcumin changed ACC cell metabolism, increasing glycolytic gene expression. However, pyruvate from glycolysis was only minimally used for lactate production and the Krebs cycle (TCA). In fact, lactate dehydrogenase, extracellular acidification rate (ECAR), TCA genes and oxygen consumption rate (OCR) were reduced. We instead found an increase in Glutamic-Pyruvic Transaminase (GPT), glutamine antiport transporter SLC1A5 and glutaminase (GLS1), supporting a metabolic rewiring toward glutamine metabolism. Targeting this mechanism, curcumin effects were improved. In fact, in a low glutamine-containing medium, the growth inhibitory effects elicited by curcumin were observed at a concentration ineffective in default growth medium. Data from this study prove the efficacy of curcumin against ACC growth and progression and point to the concomitant use of inhibitors for glutamine metabolism to improve its effects.

## 1. Introduction

Adrenocortical carcinoma (ACC) is a rare endocrine neoplasm affecting the adrenal cortex. Surgical resection remains the treatment of choice, while the pharmacological interventions include monotherapy with the adrenolytic drug mitotane or its combination with other chemotherapeutic drugs. However, the response rate is very limited, and patients are at high risk of relapse and metastasis. This is probably due to the extremely complex molecular heterogeneity of adrenocortical cancer. In fact, genomic studies confirm that ACC pathogenesis is a multi-molecular event [1]. 

We have lately focused our attention on the role played in ACC by the nuclear receptor estrogen-related receptor-α (ERRα), mainly involved in bioenergetics processes and a common downstream target of multiple pathways. ERRα protein depletion caused a mitochondrial dysfunction leading to cell death [2]. Accordingly, ERRα inhibition in grafted H295R cells strongly reduced ACC growth. More recently, we have shown that targeting ERRα decreases ACC cell motility by profoundly affecting ACC metabolism [3]. In fact, ERRα belongs to the transcriptional machinery governing not only mitochondrial biogenesis but also respiratory chain capacity. A major function of mitochondria is to generate energy by utilizing different substrates. Although malignant cells undergo reprogramming of energy metabolism toward aerobic glycolysis [4], they also depend on mitochondrial oxidative metabolism for proliferation and survival. In ACC, proteomic studies evidenced that targeting ERRα reduces expression of genes related to cell metabolism and particularly to mitochondrial functions, further demonstrated by a reduced oxygen consumption rate evaluated by Seahorse analyzer. Targeting mitochondrial bioenergetics correlated with a reduction in ACC cell metastatic potential [3]. These results are in agreement with previous in vivo studies performed on leukemia [5], breast [6] and endometrial [7] tumor cells. 

Curcumin, a naturally occurring polyphenol compound found in the plant Curcuma longa, is used as an Indian spice. The therapeutic benefits of curcumin have been demonstrated in multiple pathological conditions including inflammation, metabolic syndrome, liver disease, obesity, neurodegenerative diseases and, above all, in several cancers [8]. In the latter context, curcumin modulates several intracellular targets involved in tumor progression, affecting, among others, cell cycle checkpoints, antioxidant response and apoptosis. Accumulating evidences point to mitochondria as the target for many of the beneficial effects elicited by curcumin [9]. Recently, the ability of curcumin to influence glucose metabolism and mitochondrial activity was evaluated in MCF-7 human breast cancer and CT26 colon cancer cell lines. Curcumin was able to increase glucose uptake, hexokinase activity, lactate production, and to reduce oxygen consumption, mitochondrial membrane potential and ROS generation [10]. Many of curcumin’s effects on cell metabolism seem to overlap with those observed on ACC cells following ERRα targeting [3]. Indeed, a preclinical study demonstrated that curcumin inhibition of osteosarcoma cell growth was related to a significant reduction of ERRα expression [11].

The aim of this study was to evaluate if curcumin could affect growth and motility of ACC cells by altering ERRα expression and cell metabolism.

## 2. Materials and Methods

### 2.1. Cell Cultures 

Adrenocortical cancer cells H295R and SW13 are from American Type Culture Collection (ATCC, Rockville, MD, USA) MUC-1 cells were obtained from Constanze Hantel (University of Zurich-Switzerland, Zürich, Switzerland). H295R, SW13 and MUC-1 cells were cultured as previously described [3]. Culture media for H295R and SW13 (Sigma cat. N. D6421) and MUC-1 (Gibco cat. N. 12634-010) were supplemented with 1% glutamine (Sigma G7513) corresponding to 2 mM, unless differently indicated. 

### 2.2. Western Blot Analysis

Total cell lysate was prepared in RIPA buffer (50 mM Tris-HCl, 150 mM NaCl, 1% NP-40, 0.5% sodium deoxycholate, 2 mM sodium fluoride, 2 mM EDTA, 0.1% SDS and a mixture of protease inhibitors). Western blot analysis was performed using the same amount of protein. Protein concentration was determined by the Bradford (Sigma-Aldrich, St. Louis, MO, USA) method and equal quantities were subjected to Western blot analysis. SDS-PAGE-separated proteins were electroblotted onto a nitrocellulose membrane. The blots were incubated overnight at 4 °C with the following primary antibodies: anti-ERRα (ab76228; 1:1000); anti-SR-BI (ab52629; 1:1000); anti-HMGCR (ab215365; 1:500); from Abcam, Cambridge, UK; anti-Cyclin D1 (sc-8396; 1:500), anti-PARP-1 (sc-7150; 1:2000) and anti-GAPDH (sc-32233; 1:10,000) from Santa Cruz Biotechnology, Dallas, TX, USA. Anti βactin (ab8226; 1:1000; Abcam) was used as a loading control. All antibodies were incubated with appropriate horseradish peroxidase conjugated secondary antibodies for 1 h at room temperature. Immunoreactive bands were detected by the ECL Western blotting detection system (Santa Cruz Biotechnology, sc-2048). All the whole western blot figures can be found in the Supplementary Materials.

### 2.3. Cell Viability Assays

Cell viability was measured using the 3-(4,5-dimethylthiazol-2-yl)-2,5-diphenyltetrazolium bromide (MTT) (Sigma-Aldrich) colorimetric assay, which measures mitochondrial activity in viable cells. Cells were plated in 48-well plates and, after 48 h, were treated for 24, 48, 72 h with increasing doses of curcumin (Sigma C1386) (0, 10, 20, 40, 80 µM) solubilized in DMSO, that never exceeded 0.001% *v*/*v*. For experiments performed in the presence of reduced (0.01%, 0.02 mM) and default (1%, 2 mM) glutamine concentration, cells were treated for 48 h. Fresh MTT (Sigma), resuspended in phosphate buffered saline (PBS), was added to each well (final concentration 0.33 mg/mL) and the plate was incubated at 37 °C for 2 h in an incubator humidified to 5% of CO_2_. Media were then removed, and the formazan crystals were dissolved in 200 µL of DMSO (Sigma-Aldrich). Absorbance at 570 nm was evaluated with a spectrophotometer (Synergy H1 plate reader, BioTek Instruments, Inc., Winooski, VT, USA) on treatment conditions with n = 6. Experiments were performed three times. 

### 2.4. Determination of Apoptosis by Muse Annexin V & Dead Cell Assay

Apoptosis was evaluated using the Muse Annexin V & Dead Cell Assay test. H295R, SW-13 and MUC-1 cells were plated in growth medium for 48 h in 6-well plates and then treated with 40 µM curcumin. After 24 h, cells were trypsinized, resuspended in 100 µL of reagent (~5 × 10^5^) and incubated for 20 min in the dark at room temperature. Guava^®^ Muse^®^ Cell Analyzer-Luminex instrument was used to analyze 5000 events for H295R, SW-13 and MUC-1 cells. Apoptotic cells showed annexin-V labeling. A positive control was obtained by exposing cells to UV light for two hours.

### 2.5. TUNEL Assay

The DeadEnd™ Fluorometric TUNEL System (Promega, Madison, WI, USA) kit was used to reveal DNA fragmentation after treatment with curcumin, following the manufacturer’s instructions. Cells were cultured on slides for 48 h, before being treated with curcumin (0, 40 µM) in complete medium for 48 h. At the end of the experiment, cells were washed with PBS (Phospate buffered saline) and fixed in 4% paraformaldehyde for 15 min at room temperature. Cells were then washed with PBS and permeabilized with 0.2% Triton X-100 in PBS for 5 min. After two washes with deionized water, cells were incubated with the TdT enzyme (terminal deoxynucleotidyl transferase) and with EdUTP (5-Ethynyl-2′-deoxyuridine 5′-triphosphate) for 60 min at 37 °C. After one hour, the reaction was stopped with the specific NaCl and sodium citrate solution of the kit, and after three washes with PBS the cells were stained with a solution of DAPI (4′, 6-diamidine-2-phenylindole) in order to analyze nuclear morphology. Cells were observed with a fluorescence microscope with a 200× objective.

### 2.6. RNA Extraction, Reverse Transcription and Real-Time PCR

Total RNA was extracted with PureLink™ RNA Mini Kit (Invitrogen, Carlsbad, CA, USA). One microgram of total RNA was reverse-transcribed in a final volume of 50 μL using the High Capacity cDNA Reverse Transcription Kit (Thermo Fisher, Foster City, CA, USA); cDNA was diluted 1:3 in nuclease free water. Quantitative PCR was performed using the following primer sequences:

- ERRα:

Forward 5′-CTGGTGGTTGAGCCTGAGAAG-3′

Reverse 5′-ACCACAATCTCTCGGTCAAAGAG-3′

- CYCLIN D1:

Forward 5′-CACGCGCAGACCTTCC-3′

Reverse 5′-ATGGAGGCGGATTGGAA-3′

- HK1:

Forward 5′-CAACAGCCACAGTCAAGAT-3′

Reverse 5′-AAGGAAGACCCACCAAGA-3′

- HK2:

Forward 5′-GAGTGGAGATGCACAACAA-3′

Reverse 5′-CTGGACAATGTGGTCAAAGA-3′

- PFKL:

Forward 5′-CTCATCTACGAGGGCTATGA-3′

Reverse 5′-GCTGGATGATGTTGGAGAC-3′

- LDHA:

Forward 5′-GCCGTGATAATGACCAGCTT-3′

Reverse 5′-TGGCAGCCTTTTCCTTAGAA-3′

- ACO1:

Forward 5′-ATTTAAGCCTGCTCGTGTC-3′

Reverse 5′-TTCTCTGGATCTCCTCCTAAC-3′

- ATP5F1B:

Forward 5′-TCGATCTGCTAGCTCCCTATGC-3′

Reverse 5′-GGGCTTTGGCGACATTGT-3′

- SUCLG2:

Forward 5′-GAGGCTGCTAAGAGACTAAATG-3′

Reverse 5′-TGAACACCTCCTTTCAAACC-3′

- IDH2:

Forward 5′-GCTGGAGAAGGTGTGCGTG-3′

Reverse 5′-TGTTCAGGAAGTGCTCGTTCAG-3′

- GPT:

Forward 5′-GGTGGTGGGTGAGTTATTG-3′

Reverse 5′-GTGTCTCCTTCAGCTCTTTC-3′

- SLC1A5:

Forward 5′-TGCCTTTGGGACCTCTT-3′

Reverse 5′-TTGGCCACGCCATTATTC-3

- GLS1:

Forward 5′-TCCTCAACTGGCCAAATTC-3′

Reverse 5′-CAGAAGGGAACTTTGGTATCTC-3

- 18S:

Forward 5′-CGGCGACGACCCATTCGAAC-3′

Reverse 5′-GAATCGAACCCTGATTCCCCGTC-3′

PCR reactions were performed in the QuantStudio^Tm^ 3, Real Time PCR System (Thermo Fisher) using 0.2 μM of each primer. PowerUp™ SYBR™ Green Master Mix (Thermo Fisher) with the dissociation protocol was used for gene amplification; negative controls contained water instead of first-strand cDNA. Each sample was normalized to its 18S rRNA (18S) content. Final results were expressed as n-fold differences relative to a calibrator and calculated using the ΔΔCt method.

### 2.7. Wound Healing Assay

H295R, SW-13 and MUC-1 cells were treated with increasing doses (0, 10, 20 μM) of curcumin for 24 h. Cells were grown in 6-well plates until about 80–90% confluency was reached and then a 10μL pipette tip was used to create a scratch/wound with clear edges across the width of a well. Cells were then stained with Coomassie Brilliant Blue solution (Sigma) for 10 min and photographs were acquired under an inverted phase contrast microscope (Olympus CKX53) with 10X objective at 0 h and 24 h.

### 2.8. Boyden Chamber Assay 

Cell migration was evaluated using transwell inserts (8 μm pore size, 24-well plate, Corning Costar, Cambridge, MA, USA). MUC-1 cells (4 × 10^4^/well) were treated with curcumin (0, 10 μM) and seeded in the upper chamber. Cells were incubated at 37 °C with 5% CO_2_ for 18 h (a shorter time, not sufficient to cause cell death). At the end of the experiment, cells migrated to the lower chamber were fixed and stained with a Comassie Brilliant Blue solution (Sigma) for 10 min. The migrated cells have been counted under an inverted phase contrast microscope (Olympus CKX53). 

### 2.9. Intracellular Cholesterol Extraction and Colorimetric Cholesterol Assay

Cholesterol was measured using a colorimetric cholesterol assay kit (Cell Biolabs, San Diego, CA, USA). Intracellular cholesterol was extracted from cells using a mixture of chloroform, isopropanol, and NP-40 (7:11:0.1). Purified water was then added to lysed samples, and upon centrifugation, the organic, bottom phase was taken and dried by vacuum centrifugation. The resulting lipid pellet was resuspended in 200 μL of 1× cholesterol assay buffer. Then, 50 μL of sample were processed according to the manufacturer’s instruction. 

### 2.10. Seahorse XFe96 Metabolic Flux Analysis

#### 2.10.1. Mitochondrial Stress Analysis

Real-time oxygen consumption rates (OCR) were determined using the Seahorse Extracellular Flux (XF96) analyzer (Seahorse Bioscience, Billerica, MA, USA). H295R cancer cells were seeded into XF96-well cell culture plates (Seahorse Bioscience, Billerica, MA, USA) and incubated overnight at 37 °C in a 5% CO_2_ humidified atmosphere. After 48 h, cells were treated with curcumin (10, 20 μM) for 16 h. At the end of treatment, cells were processed as previously described [3]. The obtained OCR values were normalized to the protein content within the individual wells. 

#### 2.10.2. Glycolytic Stress Analysis

The extracellular acidification rate in real time (ECAR) was determined using the Seahorse Extracellular Flux Analyzer (XF96, Seahorse Bioscience, Billerica, MA, USA). H295R cancer cells were seeded into XF96-well cell culture plates (Seahorse Bioscience, Billerica, MA, USA), and incubated overnight at 37 °C in a 5% CO_2_ humidified atmosphere. After 48 h, cells were treated with curcumin (10, 20 μM) for 16 h. At the end of treatment, cells were processed as previously described [3]. The obtained ECAR values were normalized to the protein content within the individual wells.

### 2.11. Statistical Analysis

All experiments were performed at least three times. Data are expressed as mean values ± standard error (SE). The statistical significance was analyzed using GraphPad Prism 5.0 software (GraphPad Soft-ware, Inc., San Diego, CA, USA). Groups were compared using the analysis of variance (ANOVA) with Bonferroni’s post hoc testing. Significance was defined as *p* < 0.05. 

## 3. Results

### 3.1. Curcumin Reduces Viability and Induces Apoptosis in ACC Cells

**Figure 1:**
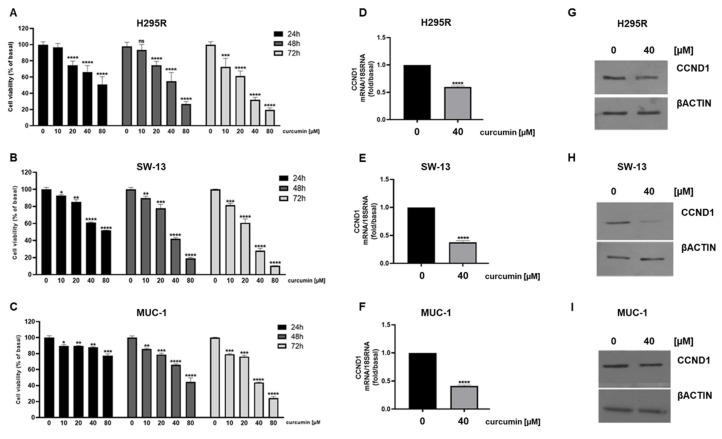
Curcumin reduces ACC cell viability. H295R (**A**), SW-13 (**B**) and MUC-1 (**C**) cells were treated with increasing doses (0, 10, 20, 40, 80 μM) of curcumin for 24, 48 and 72 h. Cell viability was evaluated by MTT assay. Results are expressed as mean ± SE of three independent experiments (* *p* < 0.05; ** *p* < 0.01; *** *p* < 0.001; **** *p* < 0.0001; ns: not significant). (**D**–**I**) Cells were treated for 24 h with curcumin. CCND1 mRNA expression was assessed by real-time RT-PCR. Data are the mean ± SE of values from at least three RNA samples (**** *p*-value < 0.0001 vs. calibrator). (**G**–**I**) CCND1 protein expression was detected by Western blotting (WB); βActin was used as a loading control. Representative WB images of three independent experiments for each cell line are shown.

We investigated the effects of curcumin on adrenocortical cancer cells proliferation. ACC cells, H295R, SW13 and MUC-1 were treated with increasing doses of curcumin for different times (24, 48 and 72 h). MTT assay demonstrated a time dependent inhibitory effect on cell viability [Figure 1A–C]. Accordingly, the expression of cyclin D1, a key cell cycle regulator governing S phase entry, was reduced by curcumin at both mRNA [Figure 1D–F] and protein [Figure 1G–I] levels. 

**Figure 2:**
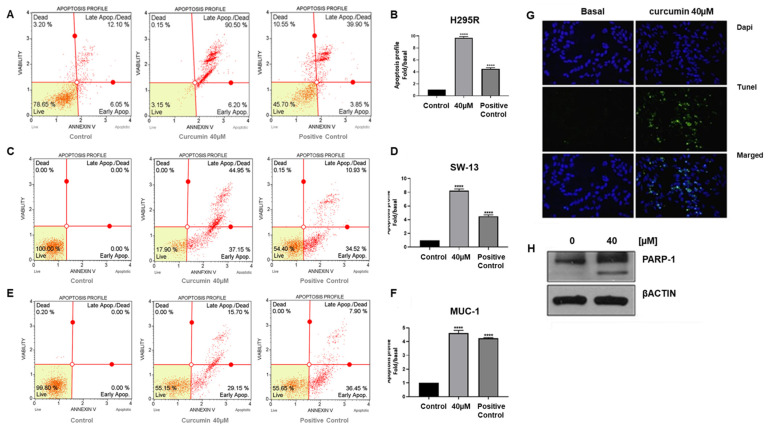
Curcumin induces apoptosis in ACC cells. H295R, SW-13 and MUC-1 cells were treated with curcumin (40 µM) for 24 h and then labeled with Annexin-V for flow cytometry analysis. Cytograms with Annexin V positive cells are shown in (**A**,**C**,**E**). Positive control is also included. (**B**,**D**,**F**) Graphical presentation of Annexin V positive cells. Data are expressed as the mean ± SE of three independent experiments, each performed in triplicate (**** *p*-value < 0.0001 vs. control). (**G**) Representative images from TUNEL assay of H295R cells treated for 48 h with curcumin; DAPI was used for nuclei staining. Magnification 200×. (**H**) Western blot detection of cleaved PARP-1 protein from H295R cells treated for 24 h with curcumin. βACTIN was used as a loading control. Blots are representative of three independent experiments with similar results.

We next verified if curcumin’s effect on cell proliferation was related to the activation of apoptosis. Therefore, ACC cells were treated with curcumin for 24 h and then labelled with Annexin V and analyzed by flow cytometry using the Muse Annexin V & Dead Cell Assay. Curcumin elicited a 9.6-fold increase in the number of apoptotic events compared to control cells [Figure 2A,B]. Similar results were observed in SW-13 and MUC-1 cells, where the apoptotic events were increased by 8.2- and 4.5-fold, respectively [Figure 2C–F]. TUNEL assay confirmed the presence of apoptotic cells following curcumin treatment [Figure 2G]. Furthermore, Western blotting analyses of cell lysates from control and treated H295R cells, demonstrated that PARP-1 (poly (ADP-ribose) polymerase-1), one of several known caspase-dependent apoptotic markers, was cleaved in response to 24 h curcumin exposure [Figure 2H].

### 3.2. Curcumin Reduces ACC Cell Motility

**Figure 3:**
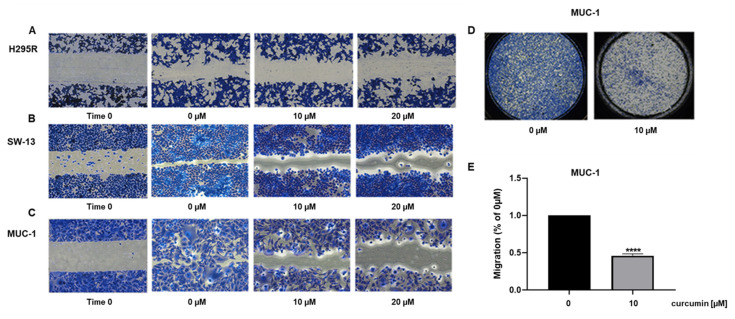
Curcumin treatment decreases ACC cell motility. Wound healing assay was performed on H295R (**A**), SW-13 (**B**) and MUC-1 (**C**) cells treated with increasing doses (0, 10, 20 μM) of curcumin for 24 h. (**D**) Images of MUC-1 migrated cells through Boyden chamber for 18 h. (Magnification 20×). (**E**) Bar graph represents the mean ± SE of cell count from three independent experiments and setting untreated cells as 100% (0 µM). **** *p* < 0.0001 vs. 0 µM.

Curcumin effects on cell motility were also investigated. ACC cell lines were treated for 24 h, with two doses of curcumin and the migratory ability was evaluated by scratch assay. Untreated cells—however, to a different extent—manifested the tendency to migrate to heal the wound, while curcumin dose-dependently prevented this effect [Figure 3A–C].

We further proved curcumin efficacy in preventing ACC cell motility by using boyden chamber assay with mitotane-resistant cell line, MUC-1, which among the used cell models, represents the most aggressive phenotype. Our results showed that after 18 h, curcumin at the dose of 10 μM was effective in preventing cell migration by 50% [Figure 3D]. Importantly, the selected times (up to 24 h) and concentrations of curcumin (10 and 20 µM) had modest effects on cell death [Figure 1], allowing to state that curcumin manifests anti-migratory effects on ACC cells.

### 3.3. Metabolic Effects of Curcumin on H295R Cells

**Figure 4:**
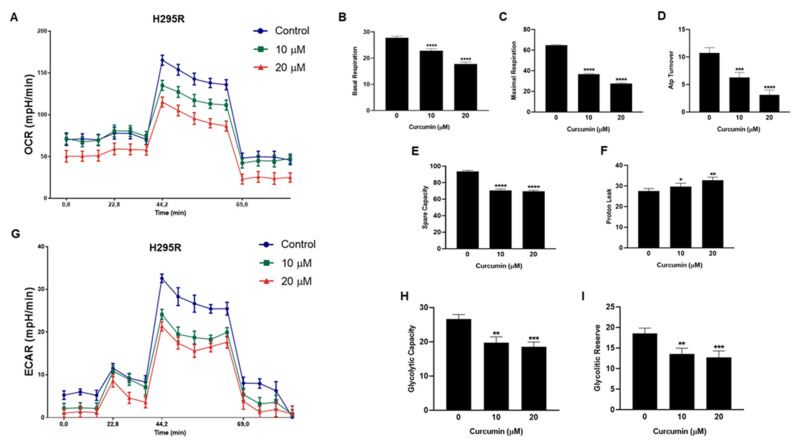
Curcumin affects H295R cell metabolism. (**A**) Real-time oxygen consumption rate (OCR) from H295R cells treated with curcumin (10 and 20 μM) for 16h assessed by Seahorse XF96 analyzer. (**B**–**F**) Bar chart showing mitochondria functional parameters: (**B**) Basal respiration; (**C**) Maximal respiration; (**D**) ATP turnover; (**E**) Spare capacity; (**F**) Proton leak. (**G**) Real-time extracellular acidification (ECAR) rates from H295R cells treated with curcumin (10 and 20μM) for 16h. Bar chart showing Glycolytic capacity (**H**) and Glycolytic reserve (**I**). * *p* < 0.05; ** *p* < 0.01; *** *p* < 0.001; **** *p* < 0.0001 vs. 0 µM.

We next assessed curcumin effects on mitochondrial respiration (OXPHOS) by using seahorse 96XF Extracellular Flux Analyzer. Specifically, H295R cells were treated with vehicle or curcumin (10 and 20 μM) for 16 h and the oxygen consumption rate (OCR) was evaluated after a sequential inhibition of the main mitochondrial energy flows. Curcumin treatment reduced OCR in a dose-dependent fashion [Figure 4A]. Additionally, curcumin significantly reduced basal respiration [Figure 4B] which represents the basal mitochondrial OXPHOS activity. Furthermore, in the presence of FCCP, an uncoupler agent, OCR was dramatically increased and sequentially blocked by the electron transfer chain (ETC) inhibitors, rotenone and antimycin A, acting on complex I and complex III, respectively [Figure 4A]. As well, curcumin markedly reduced maximal respiration [Figure 4C] in a dose dependent manner. Integrated mitochondrial electron transfer chain drives the H+ pump and powers ATP Synthase to catalyze ATP production. In H295R cells, we found that curcumin dose-dependently reduced mitochondrial ATP production [Figure 4D] while all doses of curcumin significantly reduced the Spare capacity [Figure 4E] and increased Proton leak [Figure 4F]. These data clearly indicated that curcumin suppressed mitochondrial respiration. 

The effects exerted by curcumin on glycolytic functions of H295R cells were evaluated by measuring the extracellular acidification rate (ECAR) caused by the generation of protons in the conversion of glucose to lactate during glycolysis. ECAR was measured sequentially at basal level, with glucose and in the presence of ATP synthase inhibitor (Oligomycin) and glycolysis inhibitor (2DG) with XF96 Seahorse extracellular flux analyzer and Glycolysis Stress Assay. Specifically, ECAR was evaluated in intact cells incubated with vehicle or curcumin (10 and 20 μM) for 16 h. Results clearly indicated a decrease in ECAR in the presence of curcumin [Figure 4G]. To further understand the role of curcumin in regulating aerobic glycolysis, we analyzed changes related to glycolytic capacity [Figure 4H] and glycolytic reserve [Figure 4I] that were both negatively affected by the treatments. Taken together, our findings suggest that curcumin interferes with the cellular bioenergetics of adrenocortical carcinoma.

### 3.4. Curcumin Reduces ERRα Expression and Cholesterol Content in ACC Cells

**Figure 5:**
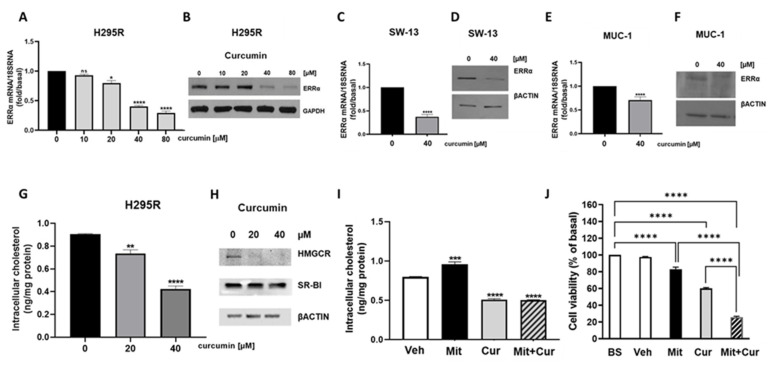
Curcumin reduces ERRα expression and Cholesterol content in ACC cells. (**A**–**F**) ACC cells were treated with the indicated doses of curcumin for 24 h and ERRα mRNA and protein expression were assessed by real-time RT-PCR (**A**,**C**,**E**) and Western blotting analysis (**B**,**D**,**F**). (**G**,**I**) Colorimetric assay was used to measure cholesterol extracted from H295R cells treated for 24h as follows: (**G**) curcumin (0, 20, 40 μM) and (**I**) vehicle (veh, DMSO), Mitotane (Mit, 10 μM), curcumin (Cur 40 μM) and their combination (Mit + Cur). (**H**) Western blotting of HMGCR and SR-BI in curcumin-treated H295R cells. (**J**) H295R cells were untreated (basal, BS), treated with vehicle (veh, DMSO), Mitotane (Mit, 10 μM), curcumin (Cur 40 μM) and their combination (Mit + Cur) 48 h. Cell viability was evaluated by MTT assay. Data and images are from at least three independent experiments. (* *p* < 0.05; ** *p* < 0.01; *** *p* < 0.001; **** *p* < 0.0001 vs. 0 µM; ns: not significant).

Curcumin effects on ACC cell metabolism were very similar to what we have recently observed targeting ERRα [3], which, among others, is a target of curcumin [11]. We first evaluated curcumin dose-response effects on ERRα expression levels in H295R cells. Increasing doses of curcumin significantly down-regulated ERRα mRNA [Figure 5A] and protein [Figure 5B] content starting from 40 µM. Curcumin treatment reduced ERRα expression also in SW-13 [Figure 5C,D] and MUC-1 cells [Figure 5E,F]. Curcumin is effective in lowering blood lipid levels, including total cholesterol [12], and inhibits cholesterol accumulation in cultured vascular smooth muscle cells [13]. Cholesterol is ERRα endogenous ligand [14], then, we decided to investigate curcumin effects on cholesterol synthesis in ACC cells and found a significant reduction in the sterol content [Figure 5G]. Western blot analysis showed that curcumin reduced HMGCR, the rate limiting enzyme in cholesterol synthesis, without affecting cholesterol uptake demonstrated by unchanged HDL receptor, SR-BI, expression [Figure 5H]. Importantly, the preferred drug for ACC treatment, mitotane, causes an increase in circulating and intratumor cholesterol [15]. We asked if curcumin could be able to overcome such an event. Determination of cholesterol content proved curcumin efficacy in preventing mitotane-induced increase in intratumor cholesterol [Figure 5I]. When the two drugs were used in combination, they potentiated their individual effects on the inhibition of ACC cell growth, which was decreased by 75% [Figure 5J].

**Figure 6:**
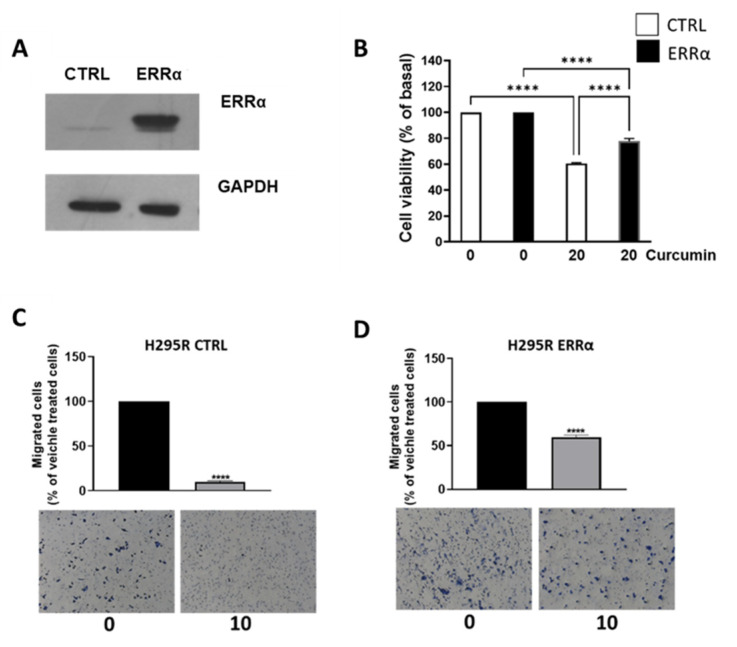
ERRα overexpression limits curcumin inhibitory effects on H295R cells. (**A**) ERRα protein expression was detected by Western blotting analysis in CTRL and ERRα overexpressing H295R cells. βActin was used as a loading control. (**B**) H295R CTRL and ERRα cells were treated with the indicated doses of curcumin for 48 h. Cell viability was evaluated by MTT assay. The dose of 0 μM curcumin (basal) was set as 100%. Results are expressed as mean ± SE of three independent experiments (*p* < 0.0001). (**C**,**D**) H295R CTRL (**C**) and ERRα (**D**) cells, treated as indicated migrated through Boyden chambers for 18 h. Images of migrated cells were acquired (Magnification 20X). Bar graphs represent the mean ± SE of cell count from three independent experiments and setting 0 µM as 100%. **** *p* < 0.0001.

To prove ERRα involvement in curcumin inhibitory effects on ACC growth and migration [Figure 1 and Figure 3], we utilized a cell line overexpressing the receptor [3]. Control (CTRL) and overexpressing cells (ERRα) were analyzed for expression of the nuclear receptor [Figure 6A] and treated for 72 h with two doses of curcumin. The growth inhibition exerted on CTRL cells was similar to parental cells, reaching a 40% decrease at the dose of 20 μM. The inhibition on ERRα overexpressing cells was less pronounced and reached 20% at the same dose [Figure 6B]. Similar effects were observed on cell migration. Migration of CTRL cells was decreased by 90% [Figure 6C], the same dose of curcumin affected ERRα overexpressing cells by a smaller extent, decreasing migration by 40% [Figure 6D]. These data proved that curcumin effects relate to its ability to decrease ERRα expression. 

### 3.5. Metabolic Genes in ACC Cells Are Affected by Curcumin

**Figure 7:**
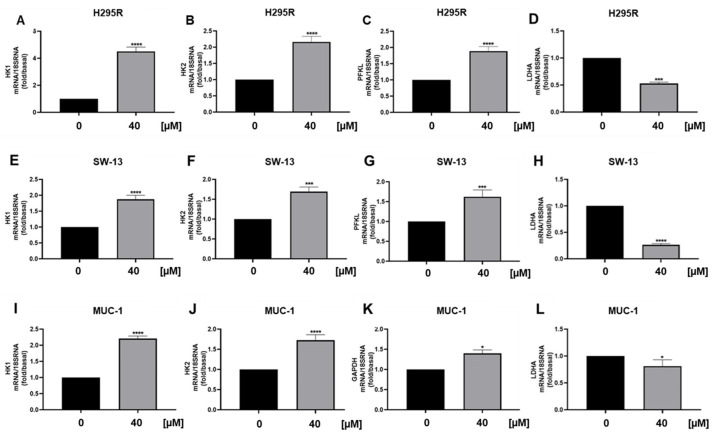
Curcumin effects on glycolytic gene expression in ACC cells. Real-time RT-PCR for HK1, HK2, PFKL, LDHA gene expression in H295R cells (**A**–**D**), SW-13 cell (**E**–**H**) and MUC-1 cells (**I**–**L**) treated with curcumin (40 μM) for 24 h. Data represent the mean ± SE of values from at least three independent RNA samples (* *p* < 0.05; *** *p* < 0.001; **** *p* < 0.0001 vs. 0 µM).

Considering the role of ERRα as the metabolic gatekeeper and transcription factor for many genes involved in glycolysis, TCA and OXPHOS [16], we evaluated the effects of curcumin on the transcription of specific metabolic genes. Glycolytic genes, HK1, HK2 and PFKL were up-regulated while LDHA was down-regulated in all three ACC cell lines [Figure 7]. 

**Figure 8:**
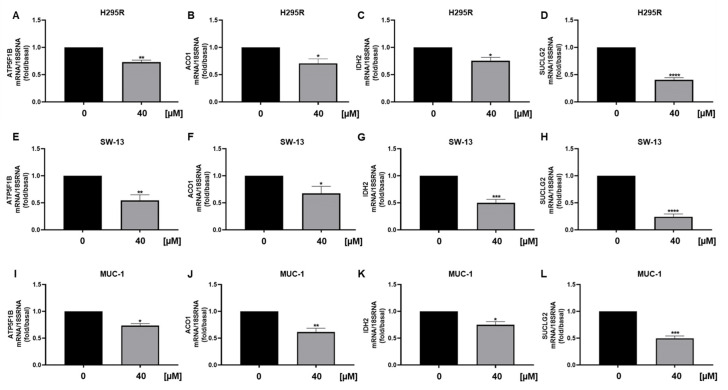
Curcumin effects on TCA gene expression in ACC cells. Real-time RT-PCR for ATP5F1B, ACO1, IDH2, SUCLG2 gene expression in H295R cells (**A**–**D**), SW-13 cell (**E**–**H**) and MUC-1 cells (**I**–**L**) treated with curcumin (40 μM) for 24 h. Data represent the mean ± SE of values from at least three independent RNA samples (* *p* < 0.05; ** *p* < 0.01; *** *p* < 0.001; **** *p* < 0. 0001 vs. 0 µM).

**Figure 9:**
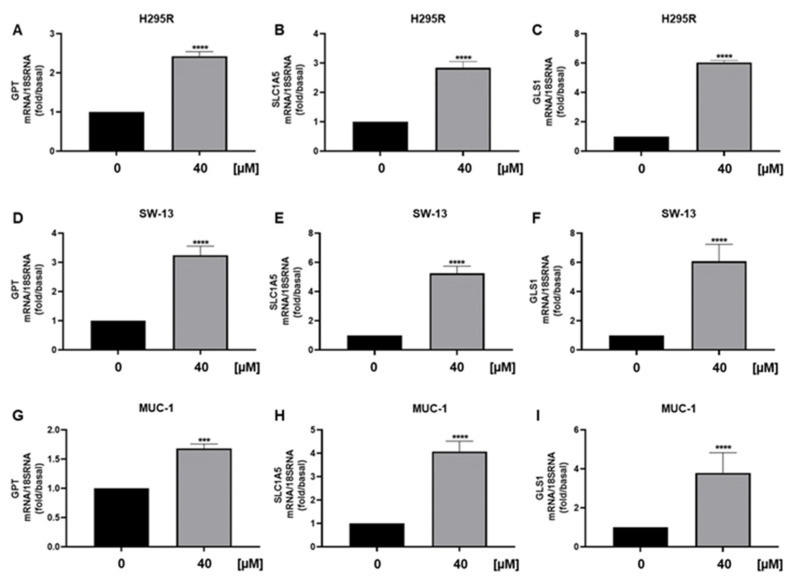
Curcumin effects on glutamine metabolism in ACC cell lines. Real-time RT-PCR for GPT, SLC1A5, GLS1 gene expression in H295R cells (**A**–**C**), SW-13 cell (**D**–**F**) and MUC-1 cells (**G**–**I**) treated with curcumin (40 μM) for 24 h. Data represent the mean ± SE of values from at least three independent RNA samples (*** *p* < 0.001; **** *p* < 0.0001 vs. 0 µM).

**Figure 10:**
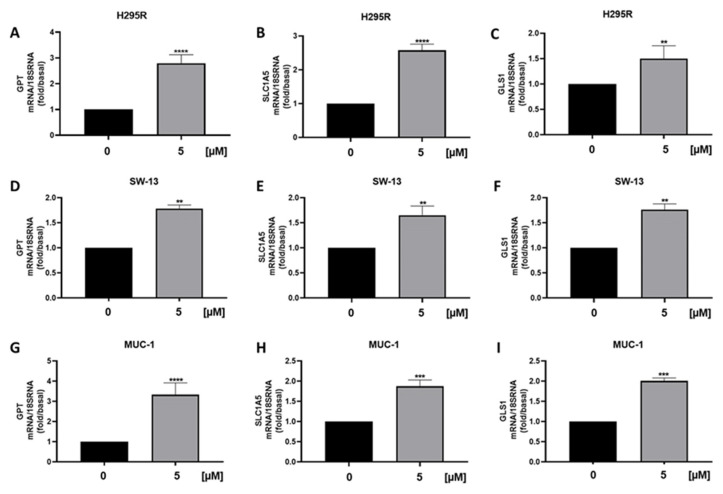
Effects of ERRα depletion on glutamine metabolism in ACC cell lines. Real-time RT-PCR for GPT, SLC1A5, GLS1 gene expression in H295R cells (**A**–**C**), SW-13 cell (**D**–**F**) and MUC-1 cells (**G**–**I**) treated with XCT790 (5 μM) for 24 h. Data represent the mean ± SE of values from at least three independent RNA samples (** *p* < 0.01; *** *p* < 0.001; **** *p* < 0.0001 vs. 0 µM).

**Figure 11:**
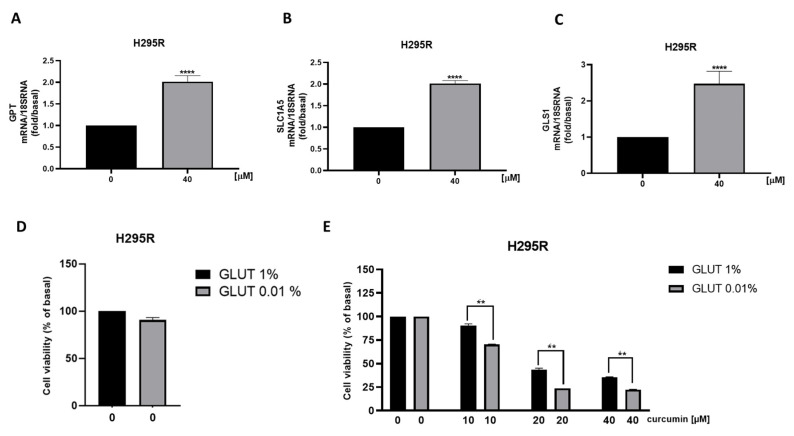
Limiting glutamine availability potentiates curcumin anti tumoral effects. (**A**–**C**) Real-time RT-PCR for GPT, SLC1A5, GLS1 gene expression in H295R cells treated for 24h with curcumin (40 μM) in the presence of low (0.01%) glutamine (GLUT) concentration. Data represent the mean ± SE of values from at least three independent RNA samples (** *p* < 0.01). (**D**,**E**) H295R cells were treated with increasing doses (0, 10, 20, 40 μM) of curcumin for 48h in the presence of default (1%) and low (0.01%) glutamine concentration. Cell viability was evaluated by MTT assay. Results are expressed as mean ± SE of three independent experiments (**** *p* < 0.01 vs. 0 µM; ** *p* < 0.01).

We also observed a reduction in ATPV mRNA [Figure 8A], a known ERRα target that we have shown decreased in ACC following the receptor targeting [3]. Furthermore, analysis of genes encoding for enzymes involved in the TCA cycle ACO1, IDH2 and SUCLG2, demonstrated a decrease in mitochondrial function [Figure 8B–D], supported by metabolic data [Figure 4A]. Similarly, SW13 [Figure 8E–H] and MUC-1 [Figure 8I–L] cells showed reduced TCA gene expressions. We then analyzed mRNA levels of GPT, needed for pyruvate conversion into alanine, which was significantly upregulated by curcumin [Figure 9A]. Alanine can be used in exchange for glutamine import through the membrane transporter SLC1A5, that we found significantly upregulated by curcumin [Figure 9B], together with glutaminase (GLS1), the glutamine catabolizing enzyme [Figure 9C]. Similar effects were observed in SW13 [Figure 9D–F] and MUC-1 [Figure 9G–I] cells. Importantly, XCT790, by decreasing ERRα expression, produced the same up-regulatory effects on the expression of GPT, SLC1A5 and GLS1 genes in all three ACC cell lines [Figure 10]. To prove cell metabolic rewiring toward glutamine metabolism, we analyzed expression of these three genes in a medium with very low glutamine content (0.01% vs. 1% glutamine). The lower glutamine availability did not affect the upregulation caused by curcumin on GPT, SLC1A5 and GLS1 expression [Figure 11A–C]. Fold induction in gene expression were similar in both 1 % [Figure 9A–C] and 0.01% [Figure 11A–C] glutamine. MTT assay demonstrated that 0.01% glutamine did not affect basal ACC cell viability [Figure 11D]. However, 0.01% glutamine enhanced curcumin inhibitory effects [Figure 11E].

## 4. Discussion

ERRα is a member of the nuclear hormone receptor superfamily primarily thought to regulate energy homeostasis through interacting with peroxisome proliferator-activated receptor γ coactivator-1s (PGC1α) and coordinately control the transcription of genes of the oxidative phosphorylation pathway. Several studies suggest that ERRα represents a novel target for cancer treatment [17]. Our recent data demonstrate that targeting ERRα by the reverse agonist XCT790 or by RNAi can reduce ACC cell growth, metastatic potential and more importantly cell metabolism [2].

Treatment of ACC cells with curcumin elicited a reduction in mitochondrial respiration parameters derived from OCR measurement by Seahorse analyzer. These data were very similar to what we observed inhibiting ACC cells with XCT790 [3]. Therefore, we investigated curcumin effects on ERRα expression, and found that, indeed, curcumin caused a significant reduction in ERRα expression and in intracellular content of cholesterol, ERRα endogenous ligand [14]. Our previous study demonstrated that the use of statins, by decreasing the intratumoral cholesterol levels, decreases ACC growth [15]. Particularly relevant is our observation that curcumin combined with mitotane was able to overcome the increase in cholesterol content caused by the latter drug [15]. Determination of cholesterol content proved curcumin efficacy in preventing mitotane-induced increase in intratumor cholesterol, offering an alternative to statins, currently used to treat the hypercholesterolemia caused by mitotane. Curcumin would add an additional beneficial effect compared to statins, since in addition to decreasing HMGCR, inhibits the activity of some steroidogenic enzymes [18], an event that would further potentiate mitotane effects. High cholesterol levels can contribute to the growth and progression of ACC through at least two mechanisms. The first involves cholesterol ability to activate ERRα, the second requires cholesterol use as a substrate for the synthesis of estradiol, a steroid hormone, able to modulate the growth of ACC [2,3,15,19]. Indeed, we evidenced that curcumin inhibits the growth of ACC cells and reduces cyclin D1 expression. A previous study reported the ability of curcumin to down-regulate ERRα expression in osteosarcoma cells (OS) causing a reduction in cyclin D1 expression and tumor growth [11]. More recently, doxorubicin and cisplatin resistant OS cells have been demonstrated to have greater levels of ERRα. Targeted inhibition of ERRα by siRNAs or XCT-790 restored the sensitivity of OS resistant cells to chemotherapy [20]. It would be interesting to see if, by decreasing ERRα expression, curcumin would have a more potent effect on these resistant cells. 

Curcumin antitumor effects have been linked to apoptosis in different cancer models [21,22,23]. Through different methods, we also showed that curcumin is able to induce apoptosis. At first, we performed Annexin V assay and demonstrated a significant increase in positive cells in response to curcumin in all three ACC cell lines. TUNEL assay further proved the presence of apoptotic cells. Ultimately, western blot analysis of poly(ADP-ribose) polymerase (PARP-1) confirmed the presence of an apoptotic mechanism. 

As seen by directly targeting ERRα by XCT790 [2] curcumin decreased the migration of ACC cells. More importantly, it was effective in preventing the migration of MUC-1 cells, representing the most aggressive available ACC cell model, characterized by the resistance to mitotane and to a wide range of therapeutics [24] and with a high metastatic potential [25]. Our data fit well with other evidences linking ERRα expression to increased ability of aggressive breast cancer cells to form lung metastases [26] and ERRα knockdown to reduced migration and invasion of endometrial [27], gastric [28] and bladder cancer [29] and non-small cell lung carcinoma [30]. ERRα is a metabolic transcription factor driving the expression of many genes involved in glycolysis, TCA, OXPHOS, thus promoting tumor progression [16]. Evaluation of glycolytic gene expressions evidenced an increase in hexokinase expression (HK1 and 2), the first glycolytic enzyme and PFKL. Instead, according to ECAR parameters, we found a reduction in LDHA, indicating that pyruvate is not fully used for lactate production. Lactate can be used in the mitochondria for fueling the TCA cycle, however, curcumin reduced mitochondrial energy production demonstrated by seahorse analysis and supported by a reduction in ATP5F1B, an ERRα target gene, Aconitase (ACO1), isocitrate dehydrogenase (IDH2) and succinyl-CoA synthetase (SUCLG2) [31]. Instead, we found an increase in GPT, the enzyme needed for the conversion of pyruvate to alanine, which can exit the cell in exchange for glutamine entry through the amino acid transporter SLC1A5. This transporter was up-regulated together with glutaminase gene (GLS1) pointing out the ability of ACC cells to counteract curcumin treatment by redirecting their metabolism toward glycolysis and glutamine utilization. This metabolic rewiring appears peculiar to ACC cells. A decrease in ECAR following curcumin treatment was evidenced in several cancer types such as gastric [31], liver [32], colon [33], breast [34] and lung [35]. However, in colorectal tumor cells, curcumin downregulated the expression and activity of hexokinase II (HKII) in a concentration-dependent manner and had little effect on other key glycolytic enzymes, PFK, PGM, and LDHA. In leukemia cells, proteomic analysis revealed that the metabolic proteins down-regulated by curcumin were typically associated to glucose metabolism, whereas those up-regulated were mostly related to mitochondrial activities [36]. In MDA-MB-453 and MCF-7 breast cancer cells curcumin caused an increase in SLC1A5 expression, and its silencing prevented glutamine entry reducing curcumin antitumor effects [37]. We can speculate that breast cancer cells use glutamine for mitochondrial anaplerosis and curcumin is able to enhance its uptake further increasing ROS production and cell death by ferroptosis. Then, SLC1A5 silencing will avoid the raise in ROS levels preventing cell death. Changes in glutamine availability are not detrimental for ACC cell growth, in fact, ACC cells grown for 48 h in a low glutamine-containing medium do not behave differently from cells grown in default medium (0.02 mM vs. 2 mM). Instead, glutamine metabolism becomes mandatory in cells exposed to curcumin, meaning that treated cells are forced to become glutamine-addicted, revealing an Achilles heel to be hit therapeutically. Glutamine is the second most abundant nutrient (after glucose) in blood circulation, concentration ranges from 0.4 to 1 mM [38]. However, tumor concentrations of glutamine as high as 5.5 mM have been reported in cancer [39]. Glutamine concentrations within ACC tissues have not been reported, however, the concentrations used in cell culture media do not appear supraphysiological. Additionally, glutamine metabolizing enzymes were upregulated by curcumin in the presence of a low concentration of the amino acid (0.01%), further confirming that the metabolic rewiring is independent from glutamine availability in the experimental conditions. To our knowledge, our study is the first indicating tumor cell ability to rewire metabolism toward glutamine use in the attempt to reduce the toxic effects caused by curcumin. These findings opened up a window of opportunity to improve curcumin effectiveness by targeting glutamine metabolism. Consistent with such an assumption, the experiments using a medium with very low concentration of glutamine enhanced the antiproliferative effects of curcumin on ACC cells. 

## 5. Conclusions

Our results clearly show that curcumin reduces ERRα expression, exerting a time and dose-dependent inhibitory effect on the growth and migration of adrenocortical carcinoma cells. These events occur in concomitance with a change in the bioenergetic status. The ability of curcumin to affect ERRα expression and cell metabolism was previously reported for other tumor types. However, our most relevant findings, still needing further investigation, concern the ability of ACC cells to adapt their metabolism to overcome curcumin effects by upregulating glutamine uptake and use. Our data highlight the possibility of improving the therapeutic efficacy of curcumin against ACC through the combination with drugs inhibiting glutamine metabolism. This combination would help to overcome the limits of curcumin poor bioavailability.

## Supplementary Materials

The following supporting information can be downloaded at: https://www.mdpi.com/article/10.3390/cancers15041050/s1, All the whole western blot figures can be found in the supplementary materials.
