# Supplementary figures and images for: Adrenocortical Carcinoma (ACC) Cells Rewire Their Metabolism to Overcome Curcumin Antitumoral Effects Opening a Window of Opportunity to Improve Treatment
Source: Cancers (Basel). 2023 Feb 7;15(4):1050. doi: 10.3390/cancers15041050 (PMC9954484; doi:10.3390/cancers15041050)

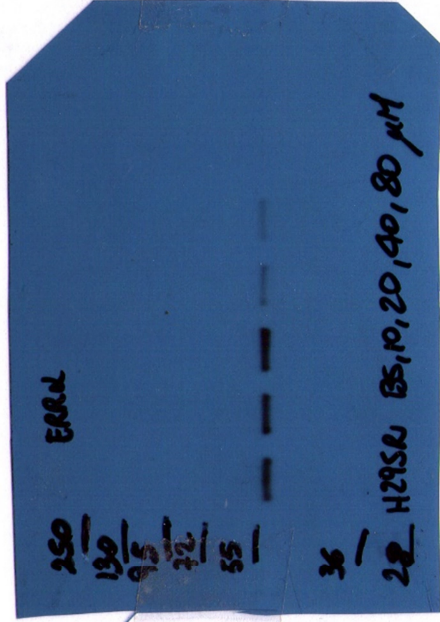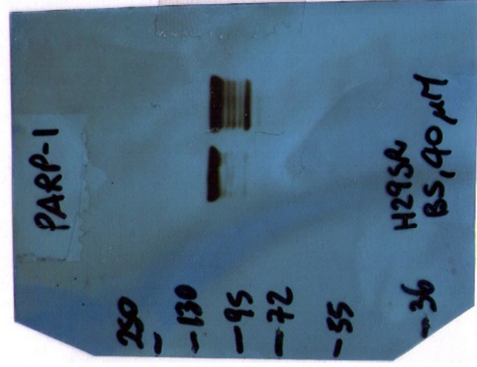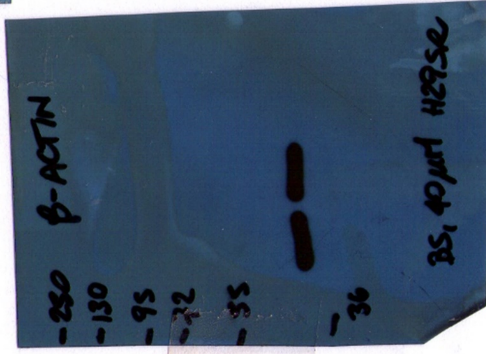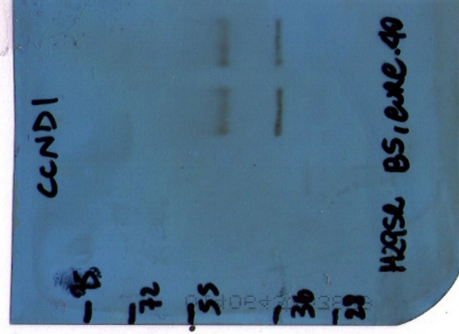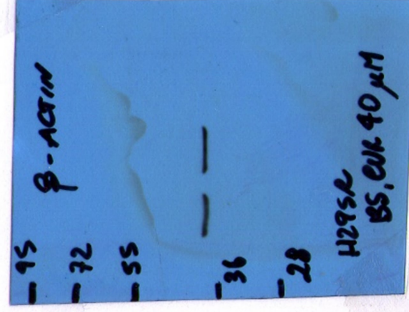

Blots SW-13

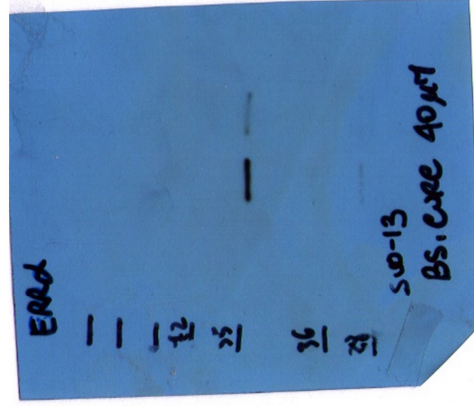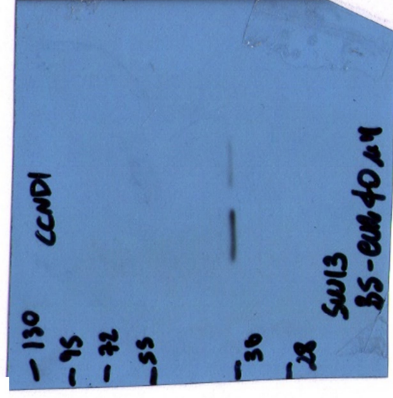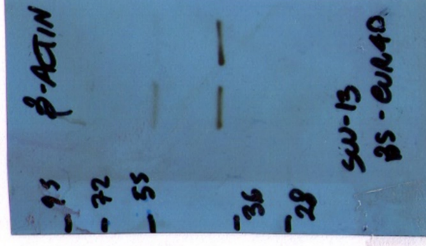

Blots MUC-1

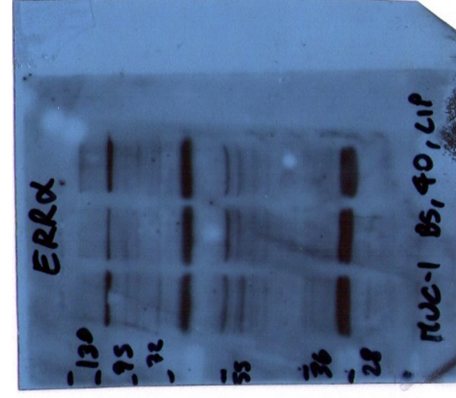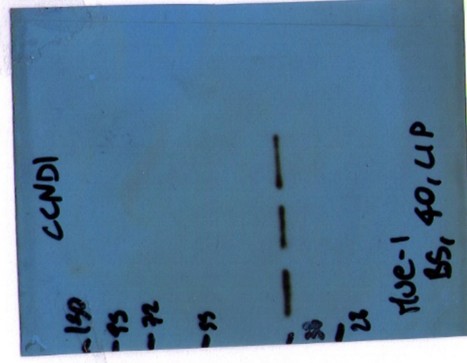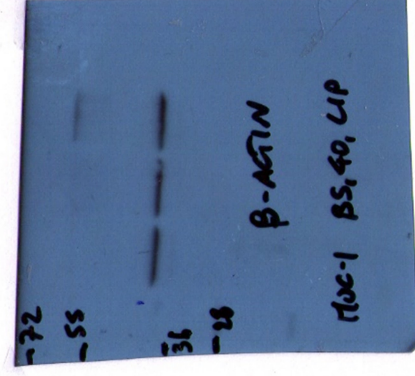

HMGCR

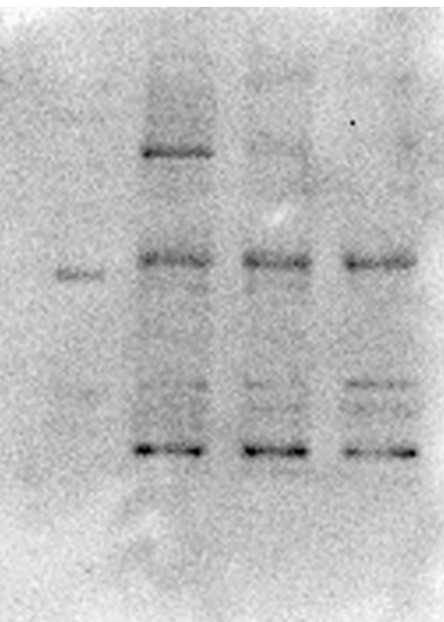

SR-BI

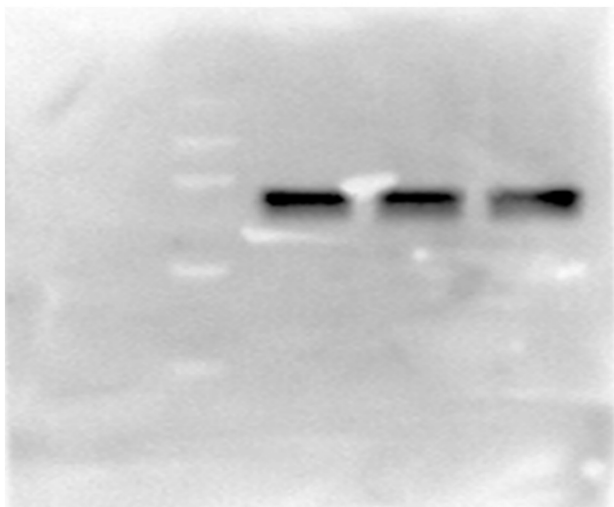

B-ACTIN

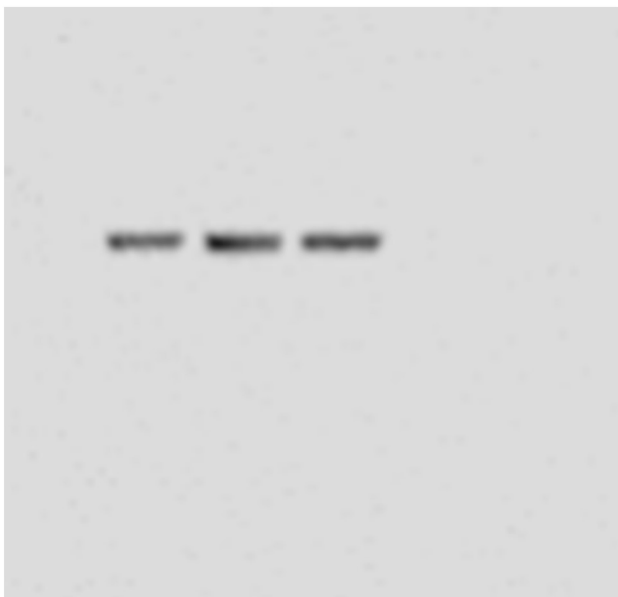

Supplement: Supplementary file 1 [file cancers-15-01050-s001.zip › cancers-2147096-supplementary.pdf]
